# Complex adaptive responses during antagonistic coevolution between *Tribolium castaneum *and its natural parasite *Nosema whitei *revealed by multiple fitness components

**DOI:** 10.1186/1471-2148-12-11

**Published:** 2012-01-26

**Authors:** Camillo Bérénos, Paul Schmid-Hempel, K Mathias Wegner

**Affiliations:** 1 Institute of Integrative Biology, Experimental Ecology, ETH Zürich Universitätstrasse 16, CHN K 12.2, 8092 Zürich, Switzerland; 2 Institute of Evolutionary Biology, University of Edinburgh, Edinburgh EH9 3JT, UK; 3 Evolutionary Ecology of Marine Fishes, Leibniz Institute for Marine Sciences (IfM-Geomar), Düsternbroker Weg 20, 24105 Kiel, Germany; 4 Alfred Wegener Institute for Polar and Marine Science, Wadden Sea Station Sylt, Hafenstrasse 43, 25992 List, Germany

## Abstract

**Background:**

Host-parasite coevolution can lead to local adaptation of either parasite or host if there is specificity (GxG interactions) and asymmetric evolutionary potential between host and parasite. This has been demonstrated both experimentally and in field studies, but a substantial proportion of studies fail to detect such clear-cut patterns. One explanation for this is that adaptation can be masked by counter-adaptation by the antagonist. Additionally, genetic architecture underlying the interaction is often highly complex thus preventing specific adaptive responses. Here, we have employed a reciprocal cross-infection experiment to unravel the adaptive responses of two components of fitness affecting both parties with different complexities of the underlying genetic architecture (i.e. mortality and spore load). Furthermore, our experimental coevolution of hosts (*Tribolium castaneum*) and parasites (*Nosema whitei*) included paired replicates of naive hosts from identical genetic backgrounds to allow separation between host- and parasite-specific responses.

**Results:**

In hosts, coevolution led to higher resistance and altered resistance profiles compared to paired control lines. Host genotype × parasite genotype interactions (G_H _× G_P_) were observed for spore load (the trait of lower genetic complexity), but not for mortality. Overall parasite performance correlated with resistance of its matching host coevolution background reflecting a directional and unspecific response to strength of selection during coevolution. Despite high selective pressures exerted by the obligatory killing parasite, and host- and parasite-specific mortality profiles, no general pattern of local adaptation was observed, but one case of parasite maladaptation was consistently observed on both coevolved and control host populations. In addition, the use of replicate control host populations in the assay revealed one case of host maladaptation and one case of parasite adaptation that was masked by host counter-adaptation, suggesting the presence of complex and probably dynamically changing fitness landscapes.

**Conclusions:**

Our results demonstrate that the use of replicate naive populations can be a useful tool to differentiate between host and parasite adaptation in complex and dynamic fitness landscapes. The absence of clear local adaptation patterns during coevolution with a sexual host showing a complex genetic architecture for resistance suggests that directional selection for generality may be more important attributes of host-parasite coevolution than commonly assumed.

## Background

The antagonistic arms race between hosts and parasites is one of the most evolutionarily significant biotic interaction [1]. Parasite species might represent more than half of the known biodiversity [2] and this ubiquity paired with detrimental fitness effects, can affect host population dynamics [3,4], genetic diversity [5], biodiversity, ecosystem functioning and community structure [6]. Consequently, parasites play a key role in various evolutionary and ecological theories, and are thought to be a major factor explaining the existence of male display in birds [7], polyandry [8], and the evolution of sex [9], despite the evolutionary costs that are associated with these phenomena.

Generally speaking, it is assumed that parasites have a larger evolutionary potential, due to their larger population size, shorter generation time, and higher mutation rates [10]. Yet, sexual hosts with complex genetic architecture underlying resistance can prevent adaptation of (asexual) parasites by producing more heterogeneous offspring, which in turn could lead to the evolution of generalist parasites [11,12]. Indeed, studies reporting experimental evolution using (facultatively) sexual hosts have shown parasite maladaptation [13] or a mosaic of patterns, with "no adaptation" being the most common finding [14]. This suggests that sexual hosts might stay ahead in the coevolutionary game by producing genetically diverse offspring, especially when genetic architecture of resistance traits are complex.

In spatially structured populations, sub-populations may experience different and asynchronous coevolutionary trajectories, which could lead to rapid between-population divergence [15,16]. Given a genetic basis of infectivity and resistance [17] the dynamic nature of coevolutionary interactions should temporarily lead to local adaptation of one or both antagonists [10,18-24]. As a consequence of local adaptation of one antagonist, local maladaptation of the other (Table 1) is also commonly observed [25-27] and is indeed predicted by the geographic mosaic theory of coevolution [28,29]. Few studies using multicellular hosts have been able to separate the two [14], as ideally, identical but naive host and/or parasite populations are needed to unequivocally interpret results obtained with cross infection studies (Table 1).

**Table 1:** Possible outcomes (rows, columns) of a cross-infection experiment where parasite performance is assayed, both, on coevolved host populations, and on control host populations paired for the same original host lines.

			Control host populations	
	Outcomes:	Mortality in matching combinations > non-matching combinations	Mortality in matching combinations < non-matching combinations	No difference between matching and non-matching combinations
	Mortality in matching combinations > non-matching combinations	1: Parasite adaptation	2: Parasite maladaptation < host maladaptation	3: Host maladaptation
**Coevolved host populations**	Mortality in matching combinations < non-matching combinations	4: Parasite adaptation < host adaptation	5: Parasite maladaptation	6: Host adaptation
	No difference between matching and non-matching combinations	7: Parasite adaptation = host adaptation	8: Parasite maladaptation = host maladaptation	9: No adaptation

The numbered entries refer to the interpretation for each possible combination of outcomes ^a^. A matching combination refers to hosts and parasite being from the same experimental coevolution replicate; non-matching otherwise.

^a ^Differentiating between parasite adaptation and host maladaptation can be facilitated if parasite fitness is assayed on replicate host populations of identical genetic background as their coevolving antagonists. In this table we have used host mortality as a measure of both parasite and host performance, as these traits are very closely linked to fitness in both antagonists in our study system [39,41]. For other study systems, relevant traits may differ. This experimental approach has, to our knowledge, never been used, but could most readily be performed in a laboratory setting.

One requirement for local adaptation in such cross-infection studies is that the outcome of exposure relies on strong host genotype (G_H_) × parasite genotype (G_P_) interactions (G_H _xG_P_). These interactions are typically suggestive of only a few underlying genes and low genetic diversity in infecting inocula [17]. However, evidence for a more complex genetic architecture underlying resistance is accumulating [30,31] and in natural populations, multiple infections seem to be the rule rather than the exception [32]. Both processes are expected to lead to less noticeable G_H _× G_P _interactions. The importance of genetic architecture is clearly illustrated by genome-wide association studies which repeatedly demonstrate that seemingly highly heritable human diseases can insufficiently be explained by genetic markers, even when taking into account the joint effects of many tens of loci [33,34]. As theory predicts that the extent to which antagonists are able to adapt decreases with increasing number of loci involved in the interaction [35], this added complexity could explain why a substantial proportion of studies fail to demonstrate clear-cut patterns of local adaptation [10]. Consequently theory predicts that in a population where host genetic variation in resistance is large, parasites may be trapped in the middle of the host phenotypic distribution, thereby leading to a reduction in parasite variance, and a generalist strategy [36].

We have already tested several predictions of the coevolutionary theory using experimental coevolution of *Tribolium castaneum *and its natural, obligately killing parasite, *Nosema whitei *[37] demonstrating coevolutionary change of both parties within local demes by time shift experiments [38,39], which represents a crucial prerequisite for local adaptation. Here, by using hosts and parasites from the same long-term study as described in references [38-40], we now tried to assess in more detail whether the reciprocal phenotypic changes led to local adaptation/specificity of either antagonist. In detail we had the following objectives: First of all, we determined whether coevolution actually leads to global and/or population-specific local adaptation patterns by performing a fully reciprocal cross infection experiment. Then, to determine the influence of trait specific genetic architecture we focused on two fitness measures with different degrees of complexity in genetic architecture [31]: (i) Host mortality (55 days post exposure) is a proxy both for host fitness and parasite fitness, as parasite transmission is only possible after host death [41,42], and shows a complex genetic architecture; and (ii) spore load, which is a measure of both transmission potential of the parasite and host resistance [39,41] and shows a rather simple genetic architecture. By cross-infecting replicate host lines from both the coevolved treatment and their paired control lines from the same genetic background kept under parasite free conditions, we increase the power to discriminate between adaptive responses of the parasite and the host, respectively. For example, if adaptation of a parasite to its own host line is neutralized by counter-adaptation of its matched coevolved host line, the parasite's adaptive response could still be detected when exposed to their matched naïve host lines, which were kept in identical but parasite-free conditions (see Table 1 for all the possible outcomes of a cross-infection experiment as performed in this paper).

If selection on parasite infectivity leads to the evolution of generality in host use, similarity in performance between parasite isolates should reflect the respective selective background and correlate with similarity in resistance between matching host lines, because resistant host lines should select for more infective/virulent parasites. This would be compatible with a form of coevolution where genetic variance of sexually reproducing hosts in combination with the complexity of host genetic architecture can prevent specific adaptation of the parasite. Complementing the longitudinal trajectory of host-parasite coevolution in this system [38] with the spatial dimension of local adaptation will provide a better understanding of the evolution of specificity as well as the selective landscape of host-parasite coevolution.

## Results

During the experiment, larvae raised in medium with parasites had significantly higher mortality rates than those raised in control (parasite-free) medium (29% vs 3%, Treatment: F_6,1531 _= 16.7, P < 0.001), regardless of their selection regime (Control: 38% vs 3%; contrast for control vs parasite sources: z-value = -5.04, P < 0.001; Coevolved: 20% vs 3%; contrast for control vs parasite sources: z-value = -3.67, P < 0.001). Furthermore, mortality induced by parasites that had coevolved with any of the host lines (i.e. averaged over parasites from all coevolved lines pooled) was lower than mortality induced by ancestral parasites (25.4 ± 10.2% S.E. *vs*. 44.9 ± 17.7; contrast analysis, z-value = 6.27, P < 0.001). Similarly, ancestral parasites achieved higher spore loads than evolved parasites (20'040 ± 3'554 S.E. *vs*. 8'436 ± 990 S.E., contrast analysis: z-value = 4.473, P < 0.001).

### Main effects, G_HOST _x G_PARASITE _interactions and the response to selection

**Figure 1:**
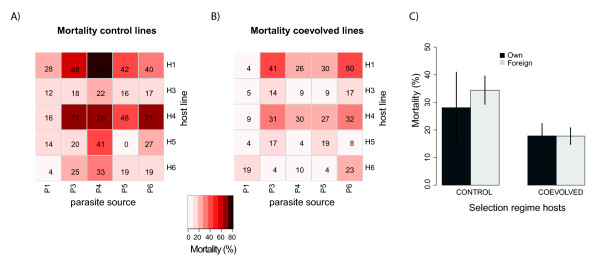
**Heatmap of mortality for all host-parasite combinations for A) the control and B) coevolved hosts separately**. Shades of red indicate the observed mortality, with darker shades corresponding to higher mortality (see legend). C) Barplot of mortality when exposed to own parasites (dark grey bars) and foreign parasites (light grey bars). Corresponding statistical details can be found in Table 1. Error bars denote ± 1 S.E.

Analyzing the cross-infection experiment for contemporary combinations (i.e. from the same generation) separately, strong main effects of host line and parasite isolate on host mortality were found (Figure 1, Table 2). Coevolved host lines were more resistant to *N. whitei*, but evolved resistance depended on the parasite isolate used (see Parasite × Selection interaction term in Table 2). Host × parasite interactions differed between coevolved and control selection regime, which is an expected result of antagonistic coevolution (Figure 1, three-way interaction in Table 2), but no significant overall host × parasite interactions were found (Figure 1, Table 2).

**Table 2:** Results of generalized linear model of host mortality after exposure to *N.whitei *using binomial error distribution. A)

Factor	Df		Deviance	Resid. Df	Resid. Dev		P(> |Chi|)	
Null deviance				1125	1276.171			
Host line	4		91.71	1121	1184.459		< 0.001	
Parasite isolate	4		45.98	1117	1138.484		< 0.001	
Selection regime	1		40.12	1116	1098.366		< 0.001	
Line:Parasite	16		8.25	1100	1090.116		0.941	
Line:Selection	4		4.73	1096	1085.386		0.316	
Parasite:Selection	4		15.41	1092	1069.976		0.004	
Line:Parasite:Selection	16		27.58	1076	1042.396		0.035	
**Results of post hoc contrast analysis of mortality**								
	Within host (coevolved lines)		Within parasite (coevolved lines)		Within host (control lines)		Within parasite (control lines)	
Line/Isolate	Z value	P	Z value	P	Z value	P	Z value	P
1	-2.54	0.049	-0.65	0.974	-2.65	0.039	2.03	0.194
3	1.81	0.272	-0.55	0.988	1.64	0.378	-1.96	0.225
4	0.12	0.999	2.14	0.153	2.93	0.016	2.38	0.084
5	0.56	0.969	0.58	0.948	-1.19	0.686	-0.03	1.000
6	2.75	0.028	-0.08	1.000	-1.38	0.551	-1.72	0.361

Levels of significance for the GLM-model fits were tested using analysis of deviance with chi-square distribution. Post hoc test results for contrasts between sympatric and allopatric combinations are shown below. Positive Z values indicate combinations where local antagonists show higher mortality than foreign antagonists.

**Figure 2:**
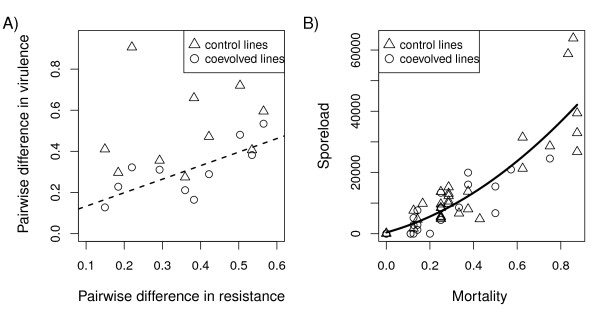
**Correlations between fitness measures**. A) Correlation between mean mortality in the eight randomly sampled beetles in each experimental block, and mean spore load as a response variable. The solid lines shows the best fitting model (R^2 ^= 0.80, F_2,57 _= 117.01, P < 0.001). Overall, there was a significant correlation between spore load and sampled mortality (Spearman rank correlation, r = 0.89, P < 0.001). B) Association of host (virulence) and parasite (failure due to resistance or infectivity) mortalities. The pairwise differences between host populations (virulence) are plotted against the respective pairwise differences in the parasite populations. Parasite population differences correlated with coevolved host differences, but only for coevolved (broken line; Mantel test, Coevolved hosts: r = 0.74, P = 0.009) and not for control hosts (r = 0.16, P = 0.308). Shown is the best fitting linear model for the coevolved hosts. s

Mortality of the beetles collected (in blocks) for measuring spore loads correlated with mortality in the survival experiment (Spearman rank correlation, r = 0.81, P < 0.001). Not surprisingly, both mortality in the experiment and sampled mortality correlated with spore load of the randomly collected subsample (Spearman rank correlation, r = 0.74, P < 0.001, and r = 0.89, P < 0.001 respectively, Figure 2a).

**Figure 3:**
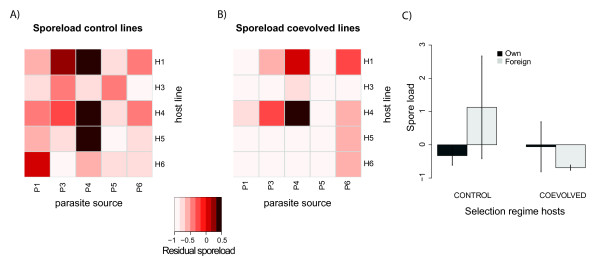
**Heatmap of residual spore load controlled for mortality for all host-parasite combinations for A) the control and B) coevolved hosts separately**. Shades of red indicate residual spore load, with lighter shades corresponding to higher residual spore load (see legend). C) Bar charts of residual spore load when exposed to own parasites (dark grey bars) and foreign parasites (light grey bars). Corresponding statistical details can be found in Table 2. Error bars denote ± 1 S.E.

When analyzing the experiment using the coevolved parasites only, spore load differed mainly between beetles that had already died or were still alive at time of collection (Table 3). Additionally, spore load differed between host lines and parasite isolates, and was generally lower in coevolved hosts, but there was also a significant host line × parasite isolate interaction (Figure 3a, Figure 3b, Table 3). The difference in spore load between coevolved and control host lines depended on the parasite isolate, but also on different host line × parasite isolate interactions under each selection regime (Figures 3a and 3b, Table 3).

**Table 3:** Results of a generalized linear model of spore load in a randomly collected subsample of eight beetles per experimental block.

Factor	Df		Deviance		Resid. Df	Resid. Dev		P(> |Chi|)
Null deviance					379	10657902		
Individual mortality (Dead/Alive)	1		8698191		378	1959711		< 0.001
factor(Line)	4		113410.1		374	1846301		< 0.001
Parasite	4		199320.9		370	1646980		< 0.001
Selection	1		77375.2		369	1569605		< 0.001
Line:Parasite	16		191614.9		353	1377990		< 0.001
Line:Selection	4		22822.19		349	1355168		0.303
Parasite:Selection	4		139426.9		345	1215741		< 0.001
Line:Parasite:Selection	16		146398.9		329	1069342		0.013
**Results of post hoc contrast analysis of spore load**								
	Within host (coevolved lines)		Within parasite (coevolved lines)		Within host (control lines)		Within parasite (control lines)	
Line/Isolate	Z value	P	Z value	P	Z value	P	Z value	P
1	-0.29	0.939	-0.07	1.000	0.48	0.983	0.60	0.982
3	0.47	0.822	0.38	0.998	-0.55	0.973	2.09	0.169
4	0.11	0.998	0.52	0.990	3.02	0.012	-1.21	0.726
5	0.03	1.000	0.04	1.000	1.28	0.618	-0.29	0.999
6	0.56	0.753	0.52	0.990	-0.61	0.961	0.06	1.000

Levels of significance of GLM model fits were tested using analysis of deviance with chi-square distribution. Post hoc test results for contrasts between sympatric and allopatric combinations are shown below. Positive Z values indicate combinations where were local antagonists show higher spore load than foreign antagonists.

### Any evidence for local adaptation?

When analyzing mortality, no global local parasite adaptation (in the sense ofhigher mortality on "own" hosts) was observed in either the coevolved (contrast analysis, z = 0.33, P = 0.932) or control regime (z = -0.029, P = 0.999, Figure 1c). When testing for population-specific host or parasite adaptation, the majority of cases revealed no significant adaptation (8 out of 10 comparisons in both the coevolved and control selection regime, Table 2). Within-parasite line tests for adaptation revealed no significant adaptation while within-host line tests revealed that 4 out of 10 combinations showed significant adaptation or maladaptation (Table 2). Both parasite adaptation (better with "own" host, positive Z-value, Table 2) and maladaptation (better with "foreign" hosts, negative Z-value, Table 2) were observed when assayed on host lines from either selection regime (Table 2).

Although sporeload measurements suggested local adaptation of the parasite (Figure 3c), we found no overall significant difference between own and foreign combinations in either the coevolved (z = 0.27, P = 0.954) or control treatment (z = -0.23, P = 0.967, Figure 3c). However, unlike host mortality, parasite adaptation with respect to spore load was only observed when parasites were exposed to control host lines (parasite isolate 4, Table 3). Of the nine possible scenarios of local adaptation (Table 1), we found evidence for scenario 7 (parasite adaptation = host adaptation) in line 4 when looking at mortality and spore load (Table 2 Table 3), scenario 5 (parasite maladaptation) in line 1 when looking at mortality, and scenario 3 (host maladaptation) in line 6 for mortality. In the remaining parasite and host lines no form of local adaptation could be detected.

### Correlation between host susceptibility and matching parasite virulence

Phenotypic differences in virulence (defined as parasite-exposed host mortality) between parasite isolates correlated with differentiation in resistance of its matching coevolved hosts in the coevolved hosts (Mantel test, r = 0.74, P = 0.009, Figure 2b). Such a correlation was absent when parasites were assayed on the control host lines (Mantel test, r = 0.16, P = 0.308). Parasite population differentiation in spore load did not correlate with host population differentiation in spore load (Mantel test, coevolved regime: r = -0.53, P = 0.976; control regime: r = -0.11, P = 0.575), possibly reflecting the stronger underlying host × parasite interactions.

Variation in mortality among host lines upon exposure to parasite isolates did not differ significantly between control and coevolution treatment (mean coefficient of variance: control: 63.8, coevolved: 68.6, pairwise t-test t = 1.33, df = 4, P = 0.253). Similarly, variation in induced host mortality among parasite isolates did not differ between selection regimes (mean coefficient of variance: control: 47.4, coevolved: 55.9; pairwise t-test, t = 0.65, df = 4, P = 0.551). Nevertheless, host and parasite population differentiation in performance were smaller in the coevolved selection regime than under control conditions (pairwise t-test on pairwise distances, parasite performance: t = -3.26, df = 9, P = 0.009; host resistance: t = -3.57, df = 9, P = 0.005) indicating a flattening of the fitness landscape during coevolution.

## Discussion

### A general lack of local adaptation after experimental coevolution

As proposed by Schulte *et al. *[14] a distinction needs to be made between "mosaic adaptation" (where only some host or parasite populations show local adaptation, while other populations show no adaptation or even maladaptation) and "local adaptation" (where hosts or parasite populations show local adaptation across a range of tested populations). Our results provide evidence for mosaic adaptation, indicating that adaptation and coevolution occurs, but we currently have no evidence for local adaptation in our experimental lines. Note that while parasite maladaptation can often be ascribed to host (counter-) adaptation, our experimental set up provides the means to separate these two explanatory mechanisms. Based on the predicted possible scenarios, one case where local combinations showed lower mortality could be ascribed to local parasite maladaptation, and one case of higher local mortality could be ascribed to host maladaptation (Table 2). One parasite isolate showed consistent adaptation to its host genetic background for both measured traits, which was not observed when looking at the coevolved combinations separately (Table 2, Table 3). In summary, our results are in concordance with the geographic mosaic theory of coevolution [15] which predicts that local adaptation may vary between demes, both spatially and temporarily, leading to a lack of an overall pattern of local adaptation. Additionally, in two out of five populations, no adaptation was detected, suggesting that these populations may be so-called "cold spots" [15,28].

### The effect of complexity of genetic architecture on G_HOST _x G_PARASITE _interactions

A substantial body of theoretical and empirical coevolutionary work is based on either the implicit or explicit assumption of high specificity in host-parasite interactions, which should "ideally" lead to negative frequency-dependent selection and spatio-temporal adaptation of antagonists [20,43,44]. Typically host × parasite (G_H _× G_P_) interactions occur in systems where only a few major genes and low genetic diversity in infecting inocula are involved in the compatibility [17]. However, more complex genetic architecture with epistatic interactions between many genes do not necessarily lead to such easily interpretable outcomes [45]. Despite the strong positive correlation between mortality and spore load, significant G_HOST _x G_PARASITE _interactions were indeed found for spore load but no such interactions were detected for mortality (Table 2, Table 3). As genetic architecture underlying mortality is more complex than that of spore load, (as host mortality is most likely a composite trait, with possible contributions of both resistance and tolerance, [31]) this can potentially explain the difference in G_H _× G_P _interactions between our fitness measures.

### Relative importance of specific and unspecific adaptation

Our results support other laboratory-based coevolutionary experiments that demonstrate very little evidence of specific local adaptation [13,14,46]. Pouillan *et al *[46] showed that if phage Φ2 was allowed to adapt to *Pseudomonas fluorescens *SBW25 specific adaptation to host genetic background was observed, while coevolution did not lead to adaptation, but rather to a broader infectivity range. This may indicate that the increased genotypic variance created by rapid reciprocal coevolutionary changes may preclude adaptation, and that local adaptation detected by field observations may be the result of directional selection for generality rather than evidence of underlying coevolution [20,47,48].

There are four additional factors that may explain the absence of local adaptation in this experiment. First, it has been shown that an increase in the number of sympatric units dramatically decreases the chance to detect local adaptation, but increases the probability to find host or parasite main effects [10]. And indeed, a substantial amount of variation in outcome of exposure to *N. whitei *was explained by such main effects in both parties (Table 1). Second, temporal oscillations in parasite infectivity potentially mask local adaptation, especially when these oscillations are not synchronized between populations [49]. Although such oscillations initially occur in this system, they dampened rapidly over time [38]. Therefore this seems not a crucial factor explaining the lack of local adaptation at the time point when this experiment was carried out. Third, a recent meta-analyses [50] showed that forces generating diversity such as mutation, migration are essential for local adaptation to occur. In the absence of such forces, erosion of standing genetic variation can decrease evolutionary potential of antagonists. In our experiment, the sexual and recombining hosts may be at an advantage compared with the asexual parasites. As no migration was allowed in our experiment, lineage sorting could have led to depletion of clonal diversity, thus precluding parasite adaptation. Finally, and most importantly, theoretical work suggests that if resistance is polygenic, such as the case for the *T.castaneum - N. whitei *interaction [31,51], selection may favour generalist parasites. Under such a scenario, within-host genetic variance is expected to increase during coevolution, while within-parasite genetic variance decreases, due to its dependency on the mean of the host phenotype distribution [36].

### A loss of parasite performance due to relaxed selection?

The predominant lack of (overall and population specific) local adaptation cannot be interpreted as a lack of coevolutionary responses of the parasites. Even though at the start of the experiment all lines were inoculated with the same spore cocktail [39], parasites rapidly differentiated in performance, while on average inducing lower host mortality upon exposure than the ancestral parasites. The null hypothesis is that diversification can be a result of genetic drift due to repeated bottlenecking. But diversification seems to be driven by the selective environment of their coevolving host genetic background, as parasite performance correlated strongly with host resistance (Figure 2A) meaning that coevolution on a low resistance background may be equal to relaxed selection. Our results thus demonstrate that susceptibility of a given host line can select for general attenuation of the parasite, a pattern that differs from parasite maladaptation [13]. Therefore we can show that coevolution may lead to adaptive changes and divergence between parasite isolates without leading to a pattern of general local adaptation. This leads to two important evolutionary implications. First, the genetic background of the host and its evolutionary potential seems crucial in determining the evolutionary trajectory of the host-parasite system. It also shows the importance of a directional selection component for generality during antagonistic coevolution giving support to previous studies in other systems [52-56]. Second, the observed loss of virulence under relaxed selection (i.e. a low resistance background) indicates that virulence may be costly in this system, and thus can be selected against if selection on growth rate is higher than selection on killing rate [39]. That differences in resistance can affect the evolution of virulence, additionally, confirms both theoretical and empirical work [57,58]. However, the correlation between resistance and virulence only manifested in parasites infecting the coevolved host lines. This different pattern of mortality caused by parasite exposure in the two selection regimes is likely a result of the weaker host × parasite interactions in coevolved hosts (see significant G_H _× G_P _x Selection interaction, Table 1).

## Conclusion

We presented evidence for rapid differentiation in infection profiles between parasite populations during antagonistic coevolution, which is likely to be driven by differences in selection coefficients imposed by differing resistance levels of coevolving host lines. At the same time, coevolution has led to increased host resistance and smaller differences in resistance profile between host lines. We did, however, not find general local adaptation in either host or parasite. By comparing parasite performance on coevolved and paired control populations we could nevertheless differentiate between adaptation and maladaptation of the antagonist and could observe likely cases of parasite adaptation, parasite maladaptation, and host maladaptation. Our results may suggest that, in this system, evolution towards generalist exploitation of hosts with complex genetic architectures is more likely than the evolution towards increased specificity, thereby explaining the lack of clear-cut patterns of local adaptation.

## Methods

### Selection experiment

All hosts and parasites used in this study originated from a coevolution experiment that has been continuously running for over three years prior to the infection experiment presented here, using *Tribolium castaneum *and its natural, microsporidian parasite *Nosema whitei*, of which the protocol is described in more detail in ref. [39]. A total of five random populations were chosen as our experimental lines for this cross-infection experiment; in particular, we used lines nr.1, 3, 4, 5 and 6 from the study of ref. [39]. All lines had a different genetic background, and thus by implication differing levels of initial resistance. At the start of the selection experiment (before generation 1), every F1 hybrid line was divided in half, and each half was subjected to one of two selection regimes. In the "coevolution" regime, lines were subjected to coevolution with the *Nosema whitei*, which led to mortalities of up to 40% [39]. In the "control" regime lines of identical origin and genetic background were maintained in the absence of parasites. In this way, half of the beetles of line 3, for example, were assigned to coevolution and the other half of line 3 to control - and so on for every line (representing the different genetic backgrounds). Population size was kept constant at 500 adult beetles in both selection regimes, by always collecting this number as breeders to initiate each following generation. The ancestral parasite used to inoculate the host lines at the start of the experiment was the same for all host lines, and consisted of a mixture (in equal proportions) of eight *Nosema whitei *isolates [39]. The regimes were maintained for a total of 16 generations since the start of the selection experiment, which has been described in more detail in [39]. After 16 generations we relaxed selection for one generation to start the cross-infection assay. Relaxed selection was achieved by rearing all lines from both treatments under control conditions (i.e. with standard flour and environmental conditions, and in the absence of parasites) to avoid potential trans-generational effects of exposure to parasites on resistance of assayed individuals in the infection experiment [59,60].

### Infection and survival experiment

In the following generation 17, after one generation of relaxation, we collected 500 unsexed adult beetles from each of these experimental lines to produce offspring that were later used in the cross-infection experiment. The resulting larval offspring were assigned randomly to the following treatments: (i) Exposure to the ancestral parasites (the same mixture of spores that was used to inoculate the coevolution experiment in generation 0); (ii) Exposure to spores from all 5 coevolved host lines collected after 17 generations of coevolution. All five host lines were subjected to all five parasite isolates in a full factorial design, meaning that for each host line there was one "sympatric" combination and four "allopatric" combinations, while each individual larvae still was only exposed to one parasite source; (iii) Controls that were not exposed to parasite spores. Freshly hatched larvae (1-2 days old) were randomly collected from a single jar for each host line, and were subsequently placed individually into glass vials (13 × 40 mm), containing 0.1 g of either parasite-inoculated flour (5 × 10^4 ^spores/gram) or parasite-free medium. A total of 1543 larvae were successfully distributed (5 replicate lines × 7 treatments × 2 selection regimes × ca. 25 larvae each). After their assignment to a treatment the larvae were kept under standard environmental conditions (24 h dark, 33°C, 70% humidity), and vials were checked for survival when the experiment was terminated (55 d after the assignment of larvae).

### Spore load measurement

From each experimental block (replicate host line × selection regime × infection treatment) we collected 8 random beetles for *N. whitei *spore load analysis. Spore load was measured using quantitative real time PCR of a 220 bp product of *N. whitei *16sRNA using methods as described in [31]. DNA was extracted using 96-well plate extraction kits (Qiagen), quantified using a Nanodrop 8000 (Thermo scientific), and diluted to 5 ng μl^-1^. Spore load was quantified in twofold for each sample, and quantification of samples with a difference of more than 1 CT between replicates were repeated, but excluded from further analysis if the difference remained higher than 1 CT. To quantify spores we used a duplicated fourfold dilution series of the same ten standard samples in every run. The highest dilution representing the detection threshold was set to the equivalent of one *N. whitei *spore. Negative controls, using ddH_2_0 instead of host DNA, were additionally used in every run.

### Statistical analyses

To test if exposure to parasites caused higher host mortality as compared to control conditions, we used a Generalized linear mixed model (*glmmPQL *from MASS package in R), with individual mortality as response, treatment (exposure/control) as fixed factor, and host line and selection regime as random factors. To test whether host mortality (binary trait: alive/dead) when exposed to coevolved parasites differed from exposure to ancestral parasites we used contrast analysis on the results of the model above. Individual survival (binary trait: alive/dead) was subsequently analyzed for all contemporary combinations using a GLM, with the factors host line, parasite source and selection regime, but all possible interactions were kept in the model. To test for directional selection on infectivity, we used Mantel tests to analyze the association between coevolved host resistance (which dictate the selection pressures on the parasites) and virulence of matching parasites when assayed on both coevolved and host lines.

To test if parasite-induced host mortality is predictive of transmission potential (spore load), a Spearman's rank correlation on mean values of each host-parasite combination was used. Spore load was subsequently analyzed for all contemporary combinations, using a GLM-model with spore load as response (quasipoisson error distribution), and individual mortality (dead/alive) as a fixed factor. Additional fixed factors were host line, parasite isolate, selection regime, with all interactions kept in the model. To compare means of total mortality and spore load of interest, we used multiple contrast analysis. Levels of significance for the GLM models were tested with analysis of deviance following a chi-square distribution. All statistical analyses were conducted with the R statistical package [61].

## Authors' contributions

CB designed and performed the experiment, analyzed the data and wrote the manuscript. PSH and KMW designed the experiment and wrote the manuscript. All authors have approved the final version.
